# Effect of fractional exhaled nitric oxide (*F*_ENO_)-based asthma management during pregnancy versus usual care on infant development, temperament, sensory function and autism signs

**DOI:** 10.1007/s00431-024-05578-4

**Published:** 2024-05-01

**Authors:** Olivia M. Whalen, Linda E. Campbell, Alison E. Lane, Frini Karayanidis, Carly A. Mallise, Alix J. Woolard, Elizabeth G. Holliday, Joerg Mattes, Adam Collison, Peter G. Gibson, Vanessa E. Murphy

**Affiliations:** 1 School of Psychological Sciences, University of Newcastle, Callaghan, Australia; 2 Healthy Minds Research Program, Hunter Medical Research Institute, Newcastle, Australia; 3 Hunter Medical Research Institute, Newcastle, Australia; 4 Olga Tennison Autism Research Centre, La Trobe University, Melbourne, Australia; 5 Population Health, Hunter New England Local Health District, Wallsend, Australia; 6 School of Medicine and Public Health, University of Newcastle, Callaghan, Australia; 7 Telethon Kids Institute, Australia & Medical School, University of Western Australia, Perth, Australia; 8 Department of Respiratory and Sleep Medicine, John Hunter Hospital, Newcastle, Australia; 9 Asthma and Breathing Research Program, Hunter Medical Research Institute, New Lambton Heights, Australia; 10 Department of Paediatric Respiratory and Sleep Medicine, John Hunter Children’s Hospital, Newcastle, Australia

**Keywords:** Asthma, Pregnancy, Postpartum, *F*_ENO_, Child development, Temperament, Sensory processing, Autism

## Abstract

Asthma during pregnancy is associated with a range of adverse perinatal outcomes. It is also linked to increased rates of neurodevelopmental conditions in the offspring. We aimed to assess whether fractional exhaled nitric oxide (*F*_ENO_)-based asthma management during pregnancy improves child developmental and behavioural outcomes compared to usual care. The Breathing for Life Trial was a randomised controlled trial that compared *F*_ENO_-based asthma management during pregnancy to usual care. Participants were invited to the developmental follow-up, the Breathing for Life Trial – Infant Development study, which followed up infants at 6 weeks, 6 months and 12 months. The primary outcomes were measured in infants at 12 months using the Bayley-III: Cognitive, Language and Motor composite scores. Secondary outcomes included Bayley-III social-emotional and adaptive behaviour scores, autism likelihood and sensory and temperament outcomes. The exposure of interest was the randomised intervention group. Two hundred and twenty-two infants and their 217 participating mothers were recruited to the follow-up; 107 mothers were in the intervention group and 113 were in the control group. There was no evidence of an intervention effect for the primary outcomes: Bayley-III cognitive (mean = 108.9 control, 108.5 intervention, *p* = 0.93), language (mean = 95.9 control, 95.6 intervention, *p* = 0.87) and motor composite scores (mean = 97.2 control, 97.9 intervention, *p* = 0.25). Mean scores for secondary outcomes were also similar among infants born to control and *F*_ENO_ group mothers, with few results reaching *p* < 0.05.

*Conclusion*: In this sample, *F*_ENO_-guided asthma treatment during pregnancy did not improve infant developmental outcomes in the first year of life.

*Trial registration*: ClinicalTrials.gov Identifier: ACTRN12613000202763.

**Table:** 

**What is Known:** *• Maternal asthma during pregnancy has been associated with increased rates of neurodevelopmental conditions in offspring, including intellectual disability and autism.*	
**What is New:** *• This is the first study to examine how managing asthma during pregnancy via a F*_ENO_*-guided algorithm or usual care affects infant developmental and behavioural outcomes. While the results of the study showed no impact of the intervention, and therefore do not support the integration of F*_ENO_*-based management of asthma in antenatal settings for optimal infant development, they do send a positive message about the implications of active asthma management during pregnancy on infant developmental outcomes.*

**Supplementary Information:**

The online version contains supplementary material available at 10.1007/s00431-024-05578-4.

## Introduction

Asthma is one of the most common chronic illnesses to affect pregnant women [1, 2], affecting about 12–13% of pregnant women in Australia [2, 3]. Maternal asthma is linked to various perinatal complications [4–8] and adverse perinatal outcomes [9–11]. However, active asthma management during pregnancy has been shown to reduce some of these risks [9, 10, 12, 13], suggesting that managing asthma during pregnancy could potentially modify its impact on pregnancy outcomes.

Maternal asthma during pregnancy has also been associated with increased rates of neurodevelopmental conditions in offspring. Some studies suggest a link between maternal asthma and an increased likelihood of autism [14] and intellectual disability [15, 16] in children. Autistic children whose mothers had immune conditions, including asthma, have been found to experience more behavioural and emotional challenges [17]. However, not all studies have found such associations [e.g. 18–21], with some reporting no negative developmental outcomes when maternal asthma is well-managed during pregnancy [22].

No studies have explored the specific effects of different asthma medications or asthma management strategies on cognitive and behavioural outcomes in children. *F*_ENO_ (fractional exhaled nitric oxide)-based asthma management is a suitable method of assessing airway inflammation in asthmatic pregnant women [23] and has been shown to significantly reduce exacerbations in asthmatic pregnant women [24] and improve the respiratory health of offspring [25, 26]. This highlights the need for more comprehensive research in this area, to identify children who may be predisposed to poorer outcomes in later childhood. Early, targeted support can be provided when it can have the most benefit to children and their families.

### Aim and hypotheses

In this study, we examined child developmental outcomes in infants born to mothers whose asthma was managed during pregnancy by a *F*_ENO_-guided algorithm (intervention group) versus those receiving standard asthma care (control group), as a substudy of the Breathing for Life Trial [BLT; 27, 28]. A range of measures was used to assess development, sensory function, temperament and likelihood for autism at three timepoints in the first year of the infant’s life: 6 weeks, 6 months and 12 months of age. We hypothesised that the infants of pregnant women in the intervention group would experience better developmental outcomes in their first year, compared to infants of pregnant women in the control group.

## Methods

### Participants

**Fig. 1:**
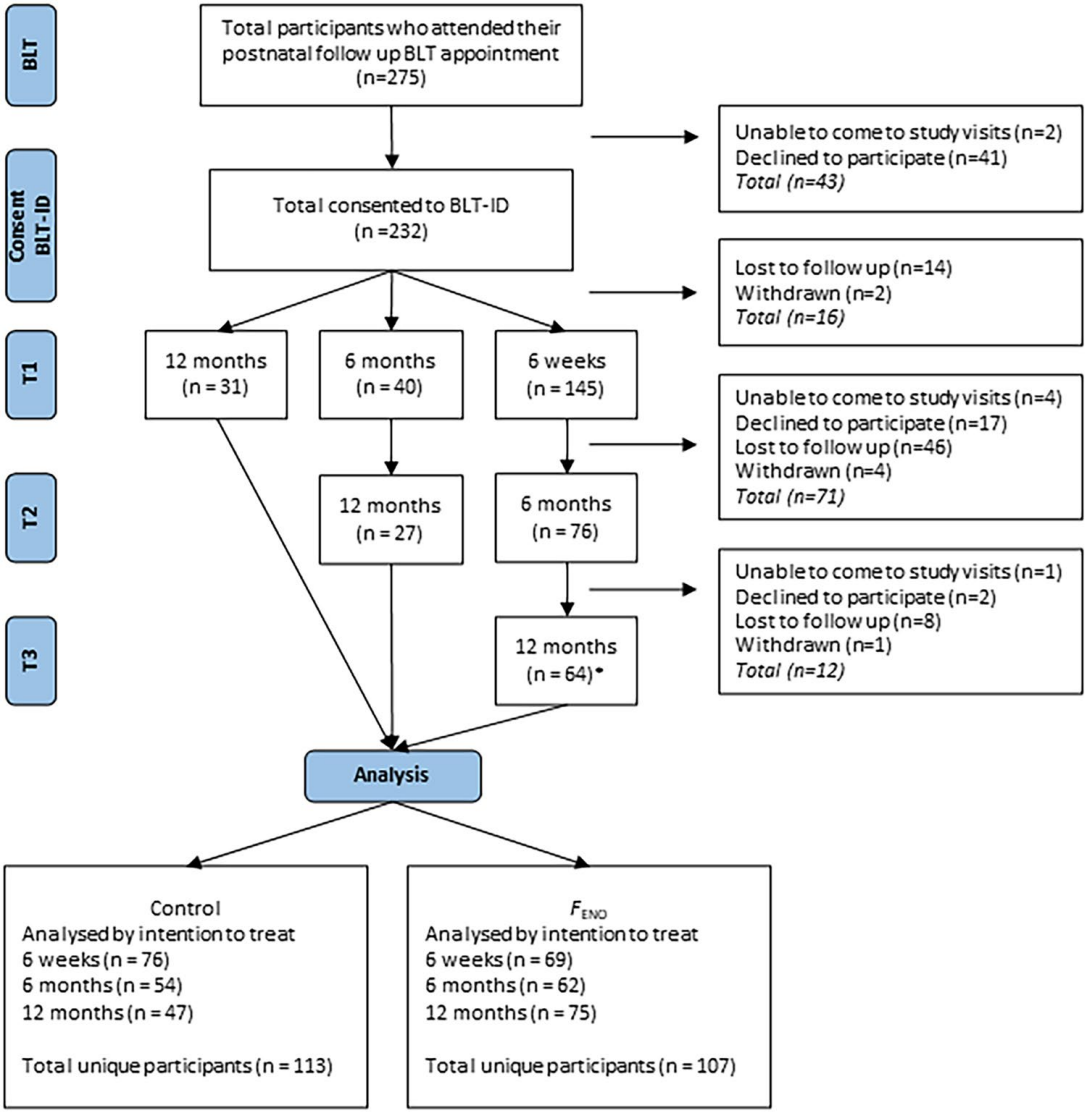
Consolidated Standards of Reporting Trials diagram. *F*_ENO_: fractional exhaled nitric oxide. Note. Not all participants from the parent Breathing for Life Trial were invited to participate in the follow-up Breathing for Life – Infant Development substudy. Only those who attended their 6 week (or 6-month or 12-month appointment) postnatal follow-up appointment for the BLT from June 2015 through to September 2018 were invited to participate in BLT-ID. *includes n = 11 participants who provided data for the 6-week and 12-month timepoints only (skipped 6-month timepoint)

Two hundred and twenty infants and their 217 participating mothers were recruited as part of the Breathing for Life Trial – Infant Development (BLT-ID) study. The BLT-ID study was a longitudinal follow-up of infants following their mother’s participation in The Breathing for Life Trial [27, 28], a randomised controlled trial (RCT) that assessed the effect of *F*_ENO_-guided asthma management during pregnancy at the John Hunter Hospital site in Newcastle, Australia. Postpartum, BLT participants were given the option of participating in the BLT-ID study during their 6-week, 6-month or 12-month BLT infant follow-up appointment. Participants could complete one, two or three sessions of the BLT-ID protocol (Fig. 1).

All participants gave written informed consent for participation. This research was conducted in accordance with the National Health and Medical Research Council (NHMRC)’s National Statement on Ethical Conduct in Human Research [29]. Ethics approval was obtained through the Hunter New England Local Health District Research ethics committee (Reference number: 15/05/20/4.05; HREC/15/HNE/164), and The University of Newcastle Human Ethics Committee (Reference number: H-2015-0307), while site-specific approval was also sought (Reference number: SSA/15/HNE/196).

#### Inclusion and exclusion criteria

For the parent trial BLT, participants were recruited from antenatal appointments at a gestational age of 12–23 weeks (supported by a dated ultrasound or clinical assessment). Women were invited to participate if they were aged over 18 years, had an asthma diagnosis from a physician, had asthma symptoms and received treatment for asthma in the last 12-months and were able to perform spirometry and *F*_ENO_ assessments. Exclusion criteria included concomitant chronic illness that may affect participation (such as heart disease), other lung diseases and drug or alcohol dependence. Further information on primary outcomes and measures of the BLT RCT have been published elsewhere [27].

### Procedure

After confirming eligibility, BLT participants were randomly allocated to either a control (asthma managed as usual) or intervention (fractional exhaled nitric oxide; *F*_ENO_-based management) group. Neither participants nor researchers were blinded to treatment group due to the differences between the intervention (regular visits during pregnancy) and usual care (one visit only). Women who were allocated to the usual care group of the trial received an asthma assessment and education on how to self-manage their asthma and did not receive any trial-specified treatment adjustment. Any changes to their treatment were managed by their general practitioner only and not the researchers. Mothers randomly allocated to receive the *F*_ENO_-based management intervention had their asthma assessed every 3–6 weeks during pregnancy and their ICS dose adjusted every 2nd visit using the *F*_ENO_ algorithm, based on their exhaled nitric oxide measure and symptoms. Regardless of intervention status, women continued their usual antenatal appointments. There was no placebo group.

The parent trial showed no significant difference between the usual care control group and the *F*_ENO_-based management intervention group on perinatal outcomes. However, in the intervention group compared to control, there was a non-significant reduction in exacerbations requiring medical intervention of 20% (OR 0.80, 95% CI 0.58, 1.10).

### Measures

#### Baseline pregnancy and birth measures

Baseline prenatal data, including maternal date of birth, estimated date of birth, postcode and smoking status were collected between 12- and 23-week gestation (Table 1). Socioeconomic status was determined via the Socioeconomic Index For Areas (SEIFA) approach, where residential postcode was used as an index of relative socioeconomic status. Participants were assigned a quintile, with lower quintiles (first, second) reflecting more socioeconomic disadvantage. Infant sex and date of birth, gestational age at birth, preterm status and birthweight information was collected from medical records.

**Table 1:** Measures used at pregnancy, birth and postpartum timepoints

	**Pregnancy**	**Birth**	**Postpartum**		
**Measure**	**Baseline**^**a**^		**6-weeks**	**6-months**	**12-months**
Demographic data					
– Maternal DOB	**✓**				
– EDB	**✓**				
– Postcode (SEIFA)	**✓**				
– Smoking status	**✓**				
Demographic data					
– Infant DOB		**✓**			
– Infant sex		**✓**			
– Gestational age at birth		**✓**			
– Birthweight		**✓**			
Demographic data					
– Maternal country of birth			**✓**		
– Primiparity			**✓**		
– Relationship status^b^			**✓**	**✓**	**✓**
– Feeding status^b^			**✓**	**✓**	**✓**
– Highest level of education (maternal & partner)^b^			**✓**	**✓**	**✓**
– Current occupation status (maternal & partner)^b^			**✓**	**✓**	**✓**
– Annual income^b^			**✓**	**✓**	**✓**
– Mental health condition^b^			**✓**	**✓**	**✓**
Sensory processing *(Sensory Profile 2)*			**✓** (ISP-2)	**✓** (ISP-2)	**✓** (TSP-2)
Temperament *(Carey Temperament Scales)*			**✓** (EITQ)	**✓** (RITQ)	**✓** (TTS)
Autism signs *(First Year Inventory 2.0)*					**✓**
Development *(Bayley Scales of Infant and Toddler Development, 3rd Edition)*				**✓** (Bayley-III Screening Test)	**✓** (Full Bayley-III Test)
Social and emotional development *(Bayley-III SEABQ)*				**✓**	**✓**

^a^Baseline refers to 12–23 weeks’ gestation

^b^Demographic information which may have changed (relationship status, current breastfeeding status, level of education, current occupation status, annual income and presence of a mental health condition) was collected again at 6 and 12-months postpartum

#### Postpartum measures

##### Demographic characteristics

At 6 weeks postpartum, further demographic data was collected via self-report including maternal country of birth, parity, relationships status, current breastfeeding (breastfed, formula fed or both), level of education and current occupation status, partner’s level of education and current occupation status, household gross annual income and presence of a mental health condition. Demographic information that could change over time (e.g. relationship status) was collected again at 6 and 12-months postpartum.

##### Clinical characteristics

Infants that were born before 37-weeks gestation were defined as being born prematurely. Infants of parents that answered “yes” to infants being either breastfed or both breastfed and formula fed were defined as being breastfed. Mothers that answered “yes” to having a mental health condition or reported taking medication for the purpose of treating a mental health condition were defined as having a mental health condition. Psychometric properties and further details for the following development measures are provided in the supplement.

##### Sensory processing

Sensory processing was assessed via the parent-report Sensory Profile 2 (SP2; [30]). Age-appropriate versions were employed: Infant Sensory Profile 2 (ISP2) at 6 weeks and 6 months and Toddler Sensory Profile (TSP2) at 12 months. Scores are produced for eight processing domains: general, auditory, visual, touch, movement, oral, behavioural (TSP2 only) and total score (ISP2 only). Higher scores reflect more sensory behaviours observed. Additionally, the TSP2 provides scores for four sensory quadrants: seeking/seeker (sensory stimulation seeking), avoiding/avoider (sensory stimulation avoidance), sensitivity/sensor (sensory event awareness) and registration/bystander (sensory event detection).

##### Temperament

Temperament was assessed using age-appropriate parent-report questionnaires from the Carey Temperament Scales (CTS); Early Infancy Temperament Questionnaire (EITQ; [31]) at 6 weeks; Revised Infant Temperament Questionnaire (RITQ; [32]) at 6 months; Toddler Temperament Scale (TTS; [33]) at 12 months. These measures yield mean scores for nine temperament domains: *activity* (motor activity; higher scores indicate more activity); *rhythmicity* (bodily function predictability; higher scores imply less predictability); *approach* (acceptance/withdrawal of novel situations, objects or people; higher scores indicate more withdrawal); *adaptability* (compliance with change; higher scores indicate slower adaptation); *intensity* (energy of responses; higher scores indicate more intense responses); *mood* (positive and negative emotions; higher scores indicate more negative mood); *distractibility* (attention diversion by stimuli; higher scores suggest less distractibility for infants under 12 months and more for those aged 12–24 months); *persistence* (the time activities are pursued and continued when distractions are present; higher scores indicate less persistence) and *threshold* (stimulus intensity needed to evoke a response; higher scores signify greater sensory sensitivity); [34]. Higher scores indicate more challenging temperament behaviours, while lower scores reflect a more manageable temperament.

##### Autism likelihood

An infant’s likelihood for autism was measured with the parent-report First Year Inventory 2.0 (FYI; [35, 36]). The FYI is a validated, general population-screening tool to identify 12-month-old infants who show characteristics of autism or related developmental conditions. Scores are calculated for the social communication and sensory regulation domains, and an overall total score. Scores ≥ 22.5 for social communication, ≥ 14.75 for sensory regulation and/or ≥ 19.2 for the total score suggest infants have an elevated likelihood for autism.

##### Cognitive, language, motor, socioemotional and adaptive behaviour development

At 6 months, infants completed The Bayley-III Scales of Infant and Toddler Development [37] screening test. The Bayley Screening test is designed to quickly identify infants at risk of delays in cognitive, language and motor development. Results are presented as raw scores for each domain and categorised as follows: “At risk” indicates potential delay, “Emerging” suggests age-appropriate skills emerging, and “Competent” signifies age-appropriate competence.

At 12 months, infants underwent the full Bayley-III developmental assessment, which includes cognitive, language (receptive and expressive) and motor (fine and gross) subscales. Performance is compared to a standardisation group, with raw scores adjusted for gestational age. Receptive and expressive language scores form a language composite, while fine and gross motor scores create an overall motor composite, both with a mean of 100 (±15). Higher scores are reflective of better developmental ability.

The parent-report Bayley-III Socioemotional and Adaptive Behaviour Questionnaire (Bayley SEABQ) were used at 6 and 12 months to gauge infants’ social and emotional development and daily living skills at home and elsewhere (adaptive behaviour). Scores cover seven skill areas for children aged under one year and 10 skill areas for children aged 1–4 years, spanning communication, community use, functional pre-academics, home living, health and safety, leisure, self-care, self-direction, social and motor skills. Like the Bayley-III assessment, performance aligns with a standardisation group. Normative composite scores have a mean of 100 (±15); higher scores are reflective of better developmental ability. These contribute to a general adaptive composite (GAC) score and scores for conceptual, social and practical domains of adaptive behaviour.

### Analysis

#### Primary outcomes

Three continuous primary outcomes were pre-specified and were all measured in infants at 12 months using the Bayley-III: cognitive composite score; language composite score and motor composite score.

#### Secondary outcomes

The following measures were obtained from the Bayley-III SEABQ at 6 months and 12 months: Social-Emotional Composite score; General Adaptive Composite (GAC) score; Adaptive Behaviour: Conceptual score; Adaptive Behaviour: Social score; Adaptive Behaviour: Practical score.

From the Bayley-III Screening test, 10 secondary outcomes were specified. The five continuous outcomes were Cognitive score; Receptive language score; Expressive language score; Fine motor score; and Gross motor score. The five categorical outcomes were: Cognitive risk (at risk/emerging/competent); Receptive language risk (at risk/emerging/competent); Expressive language risk (at risk/emerging/competent); Fine motor risk (at risk/emerging/competent) and Gross motor risk (at risk/emerging/competent).

At each timepoint, a measure of each of the following nine temperament domains was obtained (9 × 3 = 27 outcomes): Activity; Rhythmicity; Approach; Adaptability; Intensity; Mood; Persistence; Distractibility; and Threshold.

Sensory processing outcomes at 6 weeks and 6 months were measured across the following seven domains (7 × 2 = 14 outcomes): General; Auditory; Visual; Touch; Movement; Oral; and Total Processing (derived from the seven domain scores). At 12-months, scores were measured across the following seven domains: General; Auditory; Visual; Touch; Movement; Oral; Behavioural and four quadrants: Sensation Seeking; Sensation Avoidance; Sensation Sensitivity and Low Registration. The four quadrant scores are derived from the domain scores.

Three continuous measures were assessed for likelihood for autism: FYI social communication domain score; FYI sensory regulatory score and FYI total score. Three binary measures were also assessed, reflecting whether infants had a high likelihood of autism based on the FYI continuous scores: autism likelihood high (social communication score ≥ 22.5); autism likelihood high (sensory regulation score ≥ 14.75); autism likelihood high (FYI total score ≥ 19.2).

#### Exposure and explanatory variables

The exposure of interest was participation in the intervention. Potentially confounding variables included birthweight, gestational age, maternal smoking status, maternal socioeconomic status (expressed as SEIFA quintile by postcode), infant sex, preterm birth, breastfeeding and the presence of any mental health condition in the mother (Fig. S2: Directed Acyclic Graph. Supplemental data). Potential confounding of intervention effect estimates was of interest due to self-selection by mothers into this substudy, and the potential for selection bias to influence covariate balance between groups.

### Statistical analysis

Data were analysed on an intention to treat basis, by classifying infants into the intervention group their mother was randomised to. Baseline participant characteristics were summarised by intervention group using mean with standard deviation (SD) for continuous variables and frequency with percent for categorical variables.

Intervention effects were estimated using two models for each outcome. The first model adjusted only for the randomisation stratification variable of maternal smoking; the second additionally adjusted for potentially confounding variables of any breastfeeding at six weeks (yes/no), SEIFA quintile, birthweight (continuous) and preterm birth (yes/no). Since all sensitivity analyses produced similar effect estimates to the minimally adjusted model, results from the minimally adjusted model are reported here.

For continuous outcomes, the intervention effect was estimated using linear regression. Results were expressed as the least square mean difference between groups, presented as beta coefficients with 95% confidence interval (CI) and *p*-values.

Categorical Bayley-III (developmental) outcomes were converted to binary variables for analysis, combining “At risk” with “Emerging”, and comparing this combined group with “Competent”, based on low frequencies in the “At risk” category. Intervention effects for these binary outcomes were estimated using logistic regression, with results presented as odds ratios (OR) with 95% CI and *p*-values.

Given the exploratory nature of the study, a significance threshold of 0.05 was used for all outcomes. Given potential type I error inflation by multiple testing, two Bayesian quantities were also estimated and reported for each outcome: (i) Bayes factor (*BF*_01_), representing the Bayes factor in favour of the null hypothesis (*H*_0_) of no intervention effect; and (ii) the posterior probability of *H*_0_. Bayes factors were estimated using the BIC approximation and interpretation described by Wagenmakers [38], with *BF*_01_ values of 1–3, 3–20 and 20–150 considered to provide weak, positive and strong evidence in favour of *H*_0_, respectively. The posterior probability of *H*_0_ was estimated as $\frac{{BF}_{01}}{1\;+\;{BF}_{01}}$ [38] and ranges from 0–1, with higher values providing greater evidence for the null hypothesis.

Given low rates of missing data, all analyses were conducted as complete case analyses, assuming data were missing completely at random (MCAR). Data management and statistical analyses were performed using Stata V16, SAS V9.4 and JASP 0.16.3.0.

## Results

### Sample characteristics

Two hundred twenty-two infants (112 male; *N* = 145 6 weeks, *N* = 116 6-month and *N* = 122 12 months) and their 217 mothers participated in the present study. Of the 217 mothers, 213 contributed data for a single infant and 3 mothers each contributed two infants from twin births. Of the 220 infants, 107 were born to mothers randomised to the *F*_ENO_ algorithm and 113 were born to mothers randomised to the control group. Distributions for demographic and explanatory variables were similar across the two groups at each timepoint (Table 2). Sample characteristics by intervention group for key explanatory variables is presented in Supplementary data (6-month Bayley outcomes: Table 1; 12-month Bayley outcomes: Table 2).

**Table 2:** Demographic data by intervention group

**Outcome**	**Control** **M (SD)**	***F***_**ENO**_ **M (SD)**	**Total** **M (SD)**	**P Value**
Birthweight (g)	3397.9 (538.8)	3311.7 (578.5)	3353.6 (559.9)	0.26
Gestational age (w)	38.9 (1.7)	38.8 (1.7)	38.9 (1.7)	0.62

	**Control** **N (%)**	***F***_**ENO**_ **N (%)**	**Total** **N (%)**	
SEIFA quintile				
1	10 (9)	13 (12)	23 (11)	0.30
2	20 (19)	16 (15)	36 (17)	
3	43 (41)	57 (52)	100 (46)	
4	32 (30)	24 (22)	56 (26)	
5	1 (1)	0 (0)	1 (0)	
Infant sex				
Male	56 (52)	56 (50)	112 (51)	0.68
Female	51 (48)	57 (50)	108 (49)	
Preterm				
No	97 (91)	100 (88)	197 (90)	0.60
Yes	10 (9)	13 (12)	23 (10)	
Breastfed at 6-weeks				
No	20 (26)	20 (26)	40 (26)	0.92
Yes	58 (74)	56 (74)	114 (74)	
Breastfed at 6-months				
No	21 (45)	16 (34)	37 (39)	0.29
Yes	26 (55)	31 (66)	57 (61)	
Breastfed at 12-months				
No	14 (50)	26 (49)	40 (49)	0.94
Yes	14 (50)	27 (51)	41 (51)	

### Intervention effect estimates for primary outcomes

For all three primary outcomes, mean scores were similar among infants born to Control or *F*_ENO_ group mothers (control vs *F*_ENO_: cognitive 108.9 vs 108.5, coefficient (95% CI): − 0.5 (− 4.9, 3.9), *p* = 0.83; language 95.9 vs 95.6, coefficient (95% CI) =  − 0.2 (− 4.1–3.6), *p* = 0.90; motor 97.2 vs 97.9, coefficient (95% CI) = 0.7 (− 3.8–5.1), *p* = 0.77; Table 3). All Bayes factors were between 3 and 20 (range 10.55–10.91), providing “positive” evidence for the null hypothesis of no intervention effect for all outcomes. The posterior probability of H_0_ was above 0.9 for all outcomes, providing further support for all null hypotheses. Results were similar in sensitivity analyses adjusting for additional potential confounding variables (Table 3 – Supplementary data).

**Table 3:** Effect estimates for Bayley-III at 12-months (primary outcomes)

**Outcome**	**Control mean (SD)**	***F***_**ENO**_ **mean (SD)**	**Coefficient (95% CI)*F***_**ENO**_** - Control**	**P Value**	**Bayes Factor**	**Posterior probability of H**_**0**_
Cognitive Composite	108.9 (11.1)	108.5 (12.8)	-0.5 ( -4.9, 3.9)	0.83	10.81	0.92
Language Composite	95.9 (11.7)	95.6 (10.0)	-0.2 ( -4.1, 3.6)	0.90	10.91	0.92
Motor Composite	97.2 (12.8)	97.9 (12.1)	0.7 ( -3.8, 5.1)	0.77	10.55	0.91

### Secondary outcomes

#### Bayley-III outcomes at 12 months

For all five secondary outcomes measured at 12 months, mean scores were similar among infants born to Control or *F*_ENO_ group mothers (control vs *F*_ENO_: Social-Emotional Composite 99.1 vs 104.7, coefficient (95% CI) = 5.7 (− 2.8–14.2), *p* = 0.19; General Adaptive Composite 105.9 vs 104.6, coefficient (95% CI) =  − 1.0 (− 7.1–5.0), *p* = 0.74; Adaptive Behaviour: Conceptual 107.1 vs 105.7, coefficient (95% CI) =  − 1.2 (− 7.2–4.7), *p* = 0.68; Adaptive Behaviour: Social 110.4 vs 111.8, coefficient (95% CI) = 1.6 (− 4.3–7.5), *p* = 0.60; Adaptive Behaviour: Practical 99.0 vs 96.5, coefficient (95% CI) =  − 2.6 (− 8.4–3.2), *p* = 0.38; Table 4). All Bayes Factors were between 3 and 20 (range 3.75–7.48), providing “positive” evidence for the null hypothesis of no intervention effect for all outcomes. The posterior probability of H_0_ was above 0.79 for all outcomes, providing further support for all null hypotheses. Results were similar in sensitivity analyses (Table 4 – Supplementary data).

**Table 4:** Effect estimates for Bayley-III at 12-months (secondary outcomes)

**Outcome**	**Control mean (SD)**	***F***_**ENO**_ **mean (SD)**	**Coefficient (95% CI)*F***_**ENO**_** - Control**	**P Value**	**Bayes Factor**	**Posterior probability of H**_**0**_
Social-Emotional Composite	99.1 (19.9)	104.7 (17.3)	5.7 (-2.8, 14.2)	0.19	3.75	0.79
General Adaptive Composite (GAC)	105.9 (13.9)	104.6 (11.0)	-1.0 (-7.1, 5.0)	0.74	7.44	0.88
Adaptive Behaviour: Conceptual	107.1 (13.6)	105.7 (11.8)	-1.2 (-7.2, 4.7)	0.68	7.48	0.88
Adaptive Behaviour: Social	110.4 (15.7)	111.8 (10.0)	1.6 (-4.3, 7.5)	0.60	7.31	0.88
Adaptive Behaviour: Practical	99.0 (12.7)	96.5 (10.9)	-2.6 (-8.4, 3.2)	0.38	5.52	0.85

#### Bayley-III outcomes at 6 months

Most continuous outcomes showed negligible differences between groups (Table 5). Infants of *F*_ENO_ mothers scored higher on the General Adaptive Composite (GAC) than infants of Control mothers (113.2 vs 108.8, *p* = 0.05). Bayes factors were between 1 and 3 for three outcomes whose *p*-values were in the range 0.05–0.1 (General Adaptive Composite, Adaptive Behaviour: Conceptual and Adaptive Behaviour: Social), providing “weak” evidence for the null hypothesis of no intervention effect. Bayes Factors were between 3 and 20 for all other outcomes, providing “positive” evidence for the null hypothesis of no intervention effect. The posterior probability of H_0_ ranged from 0.55 to 0.91 across outcomes, providing further support for all null hypotheses. Results were similar in sensitivity analyses (Table 5 – Supplementary data) and for analyses of the categorical outcomes (Tables 6 & 7 – Supplementary data).

**Table 5:** Effect estimates for Bayley-III at 6-months (continuous)

**Outcome**	**Control mean (SD)**	***F***_**ENO**_ **mean (SD)**	**Coefficient (95% CI)*F***_**ENO**_** - Control**	**P Value**	**Bayes Factor**	**Posterior probability of H**_**0**_
Cognitive	10.7 (2.5)	10.9 (2.4)	0.2 (-0.7, 1.1)	0.63	9.58	0.91
Receptive language	7.8 (1.8)	7.9 (1.7)	0.0 (-0.6, 0.7)	0.90	10.64	0.91
Expressive language	6.8 (2.3)	6.5 (1.7)	-0.2 (-1.0, 0.5)	0.53	8.81	0.90
Fine motor	9.0 (1.5)	8.8 (1.7)	-0.3 (-0.8, 0.3)	0.39	7.41	0.88
Gross motor	9.0 (1.9)	8.9 (2.2)	-0.1 (-0.8, 0.7)	0.85	10.49	0.91
Social-Emotional Composite	105.0 (12.7)	104.8 (17.9)	0.0 (-6.6, 6.6)	0.99	9.27	0.90
General Adaptive Composite (GAC)	108.8 (13.7)	113.2 (9.6)	5.3 (0.1, 10.6)	0.05	1.25	0.55
Adaptive Behaviour: Conceptual	106.3 (14.3)	110.2 (11.9)	4.9 (-0.8, 10.7)	0.09	2.19	0.69
Adaptive Behaviour: Social	111.7 (10.9)	114.8 (8.2)	4.0 (-0.2, 8.2)	0.06	1.62	0.62
Adaptive Behaviour: Practical	105.4 (12.4)	107.3 (9.7)	2.2 (-2.7, 7.2)	0.38	6.01	0.86

#### Effect estimates for temperament and sensory outcomes and autism likelihood

All temperament outcomes across all 3 timepoints showed negligible differences between groups (Table 8 – Supplementary data). Results were similar in sensitivity analyses adjusting for additional potential confounding variables (Table 9 – Supplementary data), except for the Mood domain at 6 months, with a higher mean Mood score (indicating more negative mood) in infants of mothers randomised to *F*_ENO_ (2.8 vs 2.6, *p* = 0.03).

Results were similar for sensory outcomes across all three timepoints, with only the domain of oral processing at 6 months reaching significance with higher mean oral processing score (indicating more sensory behaviours observed) in infants of mothers randomised to the Control group (6.0 vs 5.6, *p* = 0.04; Table 10 – Supplementary data). Results were similar in sensitivity analyses adjusting for additional potential confounding variables (Table 11 - Supplementary data).

Continuous (Table 12 – Supplementary data) and binary (Table 13 – Supplementary data) outcomes of autism likelihood across all domains showed no differences between groups.

## Discussion

This study aimed to compare the developmental, temperament, sensory functioning and autism likelihood in infants from mothers who had their asthma managed by a *F*_ENO_-guided treatment algorithm to those who had their asthma managed with usual best care during pregnancy. We found no evidence of differences in development, temperament, sensory functioning and autism likelihood between infants of mothers in the two groups. This is not surprising given that there was also no evidence of any difference in a composite adverse perinatal outcome at birth between infants of mothers in the *F*_ENO_ vs the control group [27].

The Breathing for Life Trial [27, 28] is the first randomised controlled trial (RCT) to test the effect of an intervention for asthma management on perinatal outcomes. The present study, the Breathing for Life – Infant Development (BLT-ID) study is the first to compare behavioural development in infants of the above mothers. The conclusions of the primary trial were that *F*_ENO_-guided asthma intervention during pregnancy did not improve perinatal outcomes. Specifically, *F*_ENO_ -based asthma management used here measured and targeted immune activation by using an exhaled biomarker of Interleukin-13 mediated airway inflammation (exhaled nitric oxide) to assess the degree of T2 immune activation. Adjustments were made to the participant’s inhaled corticosteroid therapy to reduce *F*_ENO_ levels. Our findings that infants of mothers with asthma managed by a *F*_ENO_ algorithm do not differ significantly in temperament, sensory functioning, autism likelihood and developmental outcomes in the first year of life compared to infants of mothers with usual care. However, in the parent trial there was a non-significant, 20% reduction in exacerbations requiring medical intervention in the *F*_ENO_ group (OR 0.80, 95% CI 0.58, 1.10). It is possible that if this reduction was greater, it may have resulted in a difference in child neurodevelopmental outcomes. It is also important to consider that the embryonic period (~ gestational weeks 3–8) is a critical period for central nervous system development and neurogenesis (formation of neurons [39]). The effect of timing of environmental exposures on neurogenesis is less clear. It is therefore possible, that given the intervention was delivered in the second half of pregnancy, that it was not delivered during a critical window of neurodevelopment in pregnancy.

These findings are consistent with those of other prospective studies, which provide evidence that well-managed asthma during pregnancy is not associated with atypical developmental outcomes in children in the first few years of life [22] and are consistent with the conclusions of a recent systematic review on the subject [40]. A direct comparison between this study and Schatz [22] is not possible as they used different versions of the Bayley Scales (Bayley-I vs Bayley-III) which produce different outcome measures (mental developmental index, MDI & psychomotor developmental index, PDI vs cognitive, language and motor composite scores). Our results are consistent with a recent cross-sectional study using these data [41], that found no significant differences in sensory and temperament outcomes between children of mothers with and without asthma. Maternal asthma severity and control during pregnancy also did not affect these outcomes. However, this study compared children of mothers with and without asthma and was not an intervention study.

Importantly, we cannot comment on whether infants of mothers with untreated asthma are at an increased likelihood of autism, intellectual disability or other behavioural differences as that was not within the scope of the current study. Although there was a trend for less autism likelihood in the *F*_ENO_ group, the difference between the groups was not significant, likely due to the small numbers. It is important to note that this measure does not have perfect predictive power for later autism diagnoses, and it is possible that developmental effects do not manifest until the child is older. Studies which have assessed the longitudinal effects of asthma on child outcomes [22, 42] have not followed up infants beyond age 2 years, which leaves an important avenue for future research. The authors are currently following up this cohort on a range of developmental measures at ages 3–6. While they found no developmental differences between the intervention groups, Schatz [22] assessed 379 intervention and 376 control infants. As we assessed 107 intervention and 113 control infants, it is also possible that our sample was underpowered.

It is also worth noting that there was no placebo; instead, women were randomised into either the intervention or ‘treatment as usual’ control group. Women randomised to the control group may not have experienced typical “treatment-as-usual” asthma, given that they received self-management education and at least one prenatal asthma assessment. Meta-analyses have revealed that studies which report active management of asthma during pregnancy find no risk of adverse perinatal outcomes, such as preterm birth and neonatal hospitalisation, whereas statistically significant increased risks are found in studies which do not report active asthma management [9, 10]. This was found to be true for child developmental outcomes as well [22]. Further, asthma education during pregnancy has been shown to reduce perceptions of the teratogenic effects of medications used to treat asthma, and results in visible improvements in a woman’s inhaler technique and reduction in symptoms [43, 44]. Therefore, the control group having received an asthma assessment and self-management education during pregnancy may have changed their “as-usual” behaviour as compared to pregnant women with asthma not enrolled in the RCT.

The null findings between intervention and control groups may also be the result of trial participation. While the strengths of this study were the inclusion of participants who differed on socioeconomic status, age, ethnic background, asthma severity and level of education, as well as a proportion of women who smoked and were above a healthy weight, we have reported previously on a clear ascertainment bias in this study [45]. Given that we were able to access prenatal mental health data from hospital records, we compared the prenatal mental health of participants and non-participants of the postnatal follow-up and found that a significantly greater percentage of non-participants (31%) scored in the medium and high-risk categories of the depression measure than participants. This bias is likely to have persisted into the sample used in the present study, as we likely sampled infants of mothers with high adaptive functioning skills and help-seeking behaviours that may not be representative of the general population. It is possible that infants lost to follow-up may have mothers with more psychological distress or poorer asthma control and more severe asthma, who were not able or willing to participate in the study requirements. Alternatively, these infants could have had higher support needs or more behaviours of concern, which resulted in their mothers declining participation or withdrawing from the study. Alternatively, more participants were followed up at 12 months in the intervention group than the control, suggesting more engagement with the study. It is also possible that there is a competing effect, whereby the more intensive monitoring in this group may have contributed to higher psychological stress, potentially counteracting any beneficial effect of the intervention on developmental outcomes.

## Conclusion

While this study does not support the integration of *F*_ENO_-based management of asthma in antenatal settings for optimal infant development, it does send a positive message about the implications of asthma management during pregnancy on infant developmental outcomes, temperament, sensory processing and autism signs in the first year of life. While the findings of our study indicate that these aspects of development do not appear to be impacted, questions remain as to the long-term outcomes of these infants, and whether interventions should be implemented during the first postnatal year, where deficits are not yet apparent, to hopefully improve future outcomes.

### Supplementary Information

Below is the link to the electronic supplementary material.

Supplementary file1 (DOCX 230 KB)

## Data Availability

Data are available on request.

## Abbreviations

*F*_ENO_: Fractional exhaled nitric oxide
BLT: Breathing for Life Trial
BLT-ID: Breathing for Life Infant Development
RCT: Randomised Controlled Trial
GP: General Practitioner
SEIFA: Socioeconomic Index For Areas
ISP: Infant Sensory Profile
TSP: Toddler Sensory Profile
CTS: Carey Temperament Scales
EITQ: Early Infancy Temperament Questionnaire
RITQ: Revised Infant Temperament Questionnaire
TTS: Toddler Temperament Scale
FYI: First Year Inventory
SEABQ: Bayley-III Socioemotional and Adaptive Behaviour Questionnaire
GAC: General Adaptive Composite
M: Mean
SD: Standard deviation
CI: Confidence interval
OR: Odds Ratio
BF: Bayes factor
MCAR: Data Missing Completely at Random
MDI: Mental Developmental Index
PDI: Psychomotor Developmental Index
NHMRC: National Health and Medical Research Council

## References

[1] Clark JM, Hulme E, Devendrakumar V, Turner MA, Baker PN, Sibley CP, D’Souza SW (2007) Effect of maternal asthma on birthweight and neonatal outcome in a British inner-city population. Paediatr Perinat Epidemiol 21:154–162 17302644 10.1111/j.1365-3016.2007.00784.x

[2] Sawicki E, Stewart K, Wong S, Leung L, Paul E, George J (2011) Medication use for chronic health conditions by pregnant women attending an Australian maternity hospital. Aust N Z J Obstet Gynaecol 51:333–338 21806573 10.1111/j.1479-828X.2011.01312.x

[3] Clifton VL, Engel P, Smith R, Gibson P, Brinsmead M, Giles WB (2009) Maternal and neonatal outcomes of pregnancies complicated by asthma in an Australian population. Aust N Z J Obstet Gynaecol 49:619–626 20070710 10.1111/j.1479-828X.2009.01077.x

[4] Perlow JH, Montgomery D, Morgan MA, Towers CV, Pronto M (1992) Severity of asthma and perinatal outcome. Am J Obstet Gynecol 167:963–967 1415433 10.1016/s0002-9378(12)80020-2

[5] Wen SW, Demissie K, Liu S (2001) Adverse outcomes in pregnancies of asthmatic women: results from a Canadian population. Ann Epidemiol 11:7–12 11164114 10.1016/s1047-2797(00)00077-6

[6] Dombrowski MP, Schatz M, Wise R, Momirova V, Landon M, Mabie W, Newman RB, McNellis D, Hauth JC, Lindheimer M (2004) Asthma during pregnancy. Obstet Gynecol 103:5–12 14704237 10.1097/01.AOG.0000103994.75162.16

[7] Norjavaara E, de Verdier MG (2003) Normal pregnancy outcomes in a population-based study including 2968 pregnant women exposed to budesonide. J Allergy Clin Immunol 111:736–742 12704351 10.1067/mai.2003.1340

[8] Sobande A, Archibong E, Akinola S (2002) Pregnancy outcome in asthmatic patients from high altitudes. Int J Gynecol Obstet 77:117–121 10.1016/s0020-7292(02)00017-6 12031561

[9] Murphy V, Namazy J, Powell H, Schatz M, Chambers C, Attia J, Gibson P (2011) A meta-analysis of adverse perinatal outcomes in women with asthma. BJOG: Int J Obst Gynaecol 118:1314–1323 10.1111/j.1471-0528.2011.03055.x 21749633

[10] Murphy V, Wang G, Namazy J, Powell H, Gibson P, Chambers C, Schatz M (2013) The risk of congenital malformations, perinatal mortality and neonatal hospitalisation among pregnant women with asthma: a systematic review and meta-analysis. BJOG Int J Obst Gynaecol 120:812–822 10.1111/1471-0528.12224 23530780

[11] Lim RH, Kobzik L, Dahl M (2010) Risk for asthma in offspring of asthmatic mothers versus fathers: a meta-analysis. PLoS ONE 5:e10134 20405032 10.1371/journal.pone.0010134 PMC2853568

[12] Namazy JA, Murphy VE, Powell H, Gibson PG, Chambers C, Schatz M (2013) Effects of asthma severity, exacerbations and oral corticosteroids on perinatal outcomes. Eur Respir J 41:1082–1090 22903964 10.1183/09031936.00195111

[13] Wang G, Murphy VE, Namazy J, Powell H, Schatz M, Chambers C, Attia J, Gibson PG (2014) The risk of maternal and placental complications in pregnant women with asthma: a systematic review and meta-analysis. J Matern Fetal Neonatal Med 27:934–942 24111742 10.3109/14767058.2013.847080

[14] Croen LA, Grether JK, Yoshida CK, Odouli R, Van de Water J (2005) Maternal autoimmune diseases, asthma and allergies, and childhood autism spectrum disorders: a case-control study. Arch Pediatr Adolesc Med 159:151–157 15699309 10.1001/archpedi.159.2.151

[15] Langridge AT, Glasson EJ, Nassar N, Jacoby P, Pennell C, Hagan R, Bourke J, Leonard H, Stanley FJ (2013) Maternal conditions and perinatal characteristics associated with autism spectrum disorder and intellectual disability. PLoS ONE 8:e50963 23308096 10.1371/journal.pone.0050963 PMC3538698

[16] Leonard H, De Klerk N, Bourke J, Bower C (2006) Maternal health in pregnancy and intellectual disability in the offspring: a population-based study. Ann Epidemiol 16:448–454 16182562 10.1016/j.annepidem.2005.05.002

[17] Patel S, Dale RC, Rose D, Heath B, Nordahl CW, Rogers S, Guastella AJ, Ashwood P (2020) Maternal immune conditions are increased in males with autism spectrum disorders and are associated with behavioural and emotional but not cognitive co-morbidity. Transl Psychiatry 10:286 32796821 10.1038/s41398-020-00976-2 PMC7429839

[18] Flannery KA, Liederman J (1994) A test of the immunoreactive theory for the origin of neurodevelopmental disorders in the offspring of women with immune disorder. Cortex 30:635–646 7535215 10.1016/s0010-9452(13)80240-7

[19] Lyall K, Schmidt RJ, Hertz-Picciotto I (2014) Maternal lifestyle and environmental risk factors for autism spectrum disorders. Int J Epidemiol 43:443–464 24518932 10.1093/ije/dyt282 PMC3997376

[20] Micali N, Chakrabarti S, Fombonne E (2004) The broad autism phenotype: findings from an epidemiological survey. Autism 8:21–37 15070545 10.1177/1362361304040636

[21] Mouridsen SE, Rich B, Isager T, Nedergaard NJ (2007) Autoimmune diseases in parents of children with infantile autism: a case—control study. Dev Med Child Neurol 49:429–432 17518928 10.1111/j.1469-8749.2007.00429.x

[22] Schatz M, Harden K, Kagnoff M, Zeiger RS, Chilingar L (2001) Developmental follow-up in 15-month-old infants of asthmatic vs. control mothers. Pediatr Allergy Immunol 12:149–153 11473679 10.1034/j.1399-3038.2001.012003149.x

[23] Tamási L, Bohács A, Bikov A, Andorka C, Rigó J Jr, Losonczy G, Horváth I (2009) Exhaled nitric oxide in pregnant healthy and asthmatic women. J Asthma 46:786–791 19863281 10.1080/02770900903090004

[24] Powell H, Murphy VE, Taylor DR, Hensley MJ, McCaffery K, Giles W, Clifton VL, Gibson PG (2011) Management of asthma in pregnancy guided by measurement of fraction of exhaled nitric oxide: a double-blind, randomised controlled trial. Lancet 378:983–990 21907861 10.1016/S0140-6736(11)60971-9

[25] Mattes J, Murphy VE, Powell H, Gibson PG (2013) Prenatal origins of bronchiolitis: protective effect of optimised asthma management during pregnancy. Thorax 69:383–384 24068472 10.1136/thoraxjnl-2013-203388 PMC3963555

[26] Morten M, Collison A, Murphy V, Barker D, Meredith J, Powell H, Robinson P, Sly P, Gibson P, Mattes J (2017) Asthma Control During Pregnancy, 17Q21 Variants and Childhood-Onset Asthma. Respirol 22:100–100

[27] Murphy VE, Jensen ME, Holliday EG, Giles WB, Barrett HL, Callaway LK, Bisits A, Peek MJ, Seeho SK, Abbott A (2022) Effect of asthma management with exhaled nitric oxide versus usual care on perinatal outcomes. Eur Respir J 60 10.1183/13993003.00298-2022 PMC9669403 35777773

[28] Murphy VE, Jensen ME, Mattes J, Hensley MJ, Giles WB, Peek MJ, Bisits A, Callaway LK, McCaffery K, Barrett HL (2016) The Breathing for Life Trial: a randomised controlled trial of fractional exhaled nitric oxide (*F*_ENO_)-based management of asthma during pregnancy and its impact on perinatal outcomes and infant and childhood respiratory health. BMC Pregnancy Childbirth 16:1–10 27189595 10.1186/s12884-016-0890-3 PMC4869189

[29] NHMRC (2007) National statement on ethical conduct in human research. Canberra, NHMRC 10.1111/j.1445-5994.2011.02528.x 21762341

[30] Dunn W (2014) Sensory profile 2. Psych Corporation Bloomington, MN, USA

[31] Medoff-Cooper B, Carey WB, McDevitt SC (1993) The early infancy temperament questionnaire. J Dev Behav Pediatr 14:230–235 8408665

[32] Carey WB, McDevitt SC (1978) Revision of the infant temperament questionnaire. Pediatrics 61:735–739 662513

[33] Fullard W, McDevitt SC, Carey WB (1984) Assessing temperament in one-to three-year-old children. J Pediatr Psychol 9:205–217 6470903 10.1093/jpepsy/9.2.205

[34] Thomas A, Chess S, Birch HG, Hertzig ME, Korn S (1963) Behavioral individuality in early childhood. New York University Press

[35] Reznick JS, Baranek GT, Reavis S, Watson LR, Crais ER (2007) A parent-report instrument for identifying one-year-olds at risk for an eventual diagnosis of autism: the first year inventory. J Autism Dev Disord 37:1691–1710 17180716 10.1007/s10803-006-0303-y

[36] Turner-Brown LM, Baranek GT, Reznick JS, Watson LR, Crais ER (2013) The First Year Inventory: A longitudinal follow-up of 12-month-old to 3-year-old children. Autism 17:527–540 22781058 10.1177/1362361312439633 PMC3470769

[37] Bayley N (2006) Bayley scales of infant and toddler development. Harcourt Assessment, San Antonio, TX

[38] Wagenmakers E-J (2007) A practical solution to the pervasive problems of p values. Psychon Bull Rev 14:779–804 18087943 10.3758/bf03194105

[39] Nelson CA (2011) Neural development and lifelong plasticity. In: Keating DP (ed) Nature and nurture in early child development:45–69. Cambridge University Press

[40] Whalen OM, Karayanidis F, Murphy VE, Lane AE, Mallise CA, Campbell LE (2019) The effects of maternal asthma during pregnancy on child cognitive and behavioral development: A systematic review. J Asthma 56:130–141 29482387 10.1080/02770903.2018.1437174

[41] Mallise CA, Murphy VE, Campbell LE, Woolard AJ, Whalen OM, Milton G, Mattes J, Collison A, Gibson PG, Karayanidis F (2021) Early Sensory and Temperament Features in Infants Born to Mothers With Asthma: A Cross-Sectional Study. Front Psychol 12:713804 34690871 10.3389/fpsyg.2021.713804 PMC8531526

[42] Gordon M, Niswander KR, Berendes H, Kantor AG (1970) Fetal morbidity following potentially anoxigenic obstetric conditions: VII. Bronchial asthma. Am J Obstet Gynecol 106:421–429 5410878 10.1016/0002-9378(70)90371-6

[43] Robijn AL, Jensen ME, Gibson PG, Powell H, Giles WB, Clifton VL, Mattes J, Peek MJ, Barrett HL, Seeho SK (2019) Trends in asthma self-management skills and inhaled corticosteroid use during pregnancy and postpartum from 2004 to 2017. J Asthma 56:594–602 29716412 10.1080/02770903.2018.1471709

[44] Murphy VE, Gibson P, Talbot PI, Clifton VL (2005) Severe asthma exacerbations during pregnancy. Obstet Gynecol 106:1046–1054 16260524 10.1097/01.AOG.0000185281.21716.02

[45] Whalen OM, Campbell LE, Murphy VE, Lane AE, Gibson PG, Mattes J, Collison A, Mallise CA, Woolard A, Karayanidis F (2020) Observational study of mental health in asthmatic women during the prenatal and postnatal periods. J Asthma 57:829–841 31148493 10.1080/02770903.2019.1621888

