# Supplementary material for: Effect of fractional exhaled nitric oxide (FENO)-based asthma management during pregnancy versus usual care on infant development, temperament, sensory function and autism signs
Source: Eur J Pediatr. 2024 May 1;183(8):3199–210. doi: 10.1007/s00431-024-05578-4 (PMC11263417; doi:10.1007/s00431-024-05578-4)
Supplement: Supplementary file 1 — Supplementary file1 (DOCX 230 KB) [file 431_2024_5578_MOESM1_ESM.docx]

SUPPLEMENTARY MATERIAL

The Breathing for Life Trial – Infant Development: the effect of fractional exhaled nitric oxide (*F*_ENO_)-based management of asthma during pregnancy *versus* usual care on infant development, temperament, sensory function and autism signs

Olivia M. Whalen^1,2,3^ Linda E. Campbell^1,2,3^, Alison E. Lane^4^, Frini Karayanidis^1,2,3^, Carly A. Mallise^3,5,6^, Alix J. Woolard^7^, Elizabeth G. Holliday^3,6^, Joerg Mattes^3,6,8,9^, Adam Collison^3,6,9^, Peter G. Gibson^3,6,9,10^ & Vanessa E. Murphy^3,6,9^*

^1^ School of Psychological Sciences, University of Newcastle, Australia

^2^ Healthy Minds Research Program, Hunter Medical Research Institute, Australia

^3^ Hunter Medical Research Institute, Newcastle, Australia

^4^ Olga Tennison Autism Research Centre, La Trobe University, Melbourne, Australia

^5^ Population Health, Hunter New England Local Health District, Wallsend, Australia

^6^ School of Medicine and Public Health, University of Newcastle, Australia

^7^ Telethon Kids Institute, Perth, Australia & Medical School, University of Western Australia

^8^ Department of Respiratory and Sleep Medicine, John Hunter Hospital, Newcastle, Australia

^9^ Asthma and Breathing Research Program, Hunter Medical Research Institute, Australia

^10^ Department of Paediatric Respiratory and Sleep Medicine, John Hunter Children's Hospital, Newcastle, Australia

# Psychometric properties and details of the measures used.

**Sensory processing**: The *SP2* has good internal consistency (α = 0.60 to 0.90) and test-retest reliability (r = 0.87 to 0.97). **Temperament**: The *Carey Temperament Scales* show acceptable internal consistency (α = 0.43 to 0.86) and good test-retest reliability (r = 0.64 to 0.89). **Autism likelihood**: The *FYI* has a sensitivity of 44%, indicating that 44% percent of infants who meet the total score threshold are likely to be diagnosed with autism by age 3(1). For infants meeting both social communication and sensory regulation thresholds, 31% are likely to be diagnosed with autism, and 85% are likely to experience autism or other related developmental concerns by age 3. The FYI has a specificity of 99%, meaning that 99% of children who are not later diagnosed with autism are not flagged. The FYI has good internal consistency (α = 0.81). **Development**: *The Bayley-III Screener* includes items derived from the full Bayley-III, emphasising content quality, psychometric properties, and the ability to distinguish between high and low-performing children (clinical sensitivity). It exhibits good test-retest reliability across all ages (average reliability coefficients: .80 - .83). If an infant displayed any signs of delay in any domain, parents were offered the option to proceed with a full Bayley-III assessment for a more in-depth investigation and received guidance on enhancing their child's skills in that area. The full *Bayley-III* test isn't a diagnostic tool but quantifies functional development, screens for delays, and guides interventions. Widely used since 2006, it exhibits strong internal consistency (average reliability coefficients: .94 - .98) across all age groups for the five subscales (cognitive, expressive/receptive language, and fine/gross motor) and acceptable test-retest reliability for infants aged 9-13 months, with correlation coefficients (corrected for age) ranging from .77 to .86(2).

# Performance on Bayley-III at 12 months compared to normative data

We compared mean Bayley-III composite scores at 12 months with the normative data from the Bayley-III (M=100; SD = 15) using the Wilcoxon signed-rank test, as the normality assumption was violated. Infants across both groups scored significantly higher than the normative sample on the cognitive scale (V = 4784.00, *p* <.001, Coefficient = 0.11), but significantly lower on the language (V = 1594.50, *p* <.001, Coefficient = 0.11) and motor (V = 2558.00, *p* =.021, Coefficient = 0.13) scales (Figure 1). Importantly, however, all mean Bayley-III composite scores fell within the normative range (M=100±15).

Figure S1. Bayley-III Composite scores on the cognitive, language and motor subscales by intervention group. There were no significant differences between the two groups on any of the primary outcomes. The dotted line indicates a normative mean of 100 while the shaded area represents the normative standard deviation (±15). Error bars indicate the standard deviation.

# Sample characteristics

## 6-month developmental (Bayley) outcomes

For 6-month outcome variables (Table 1), distributions were similar across the two groups, with no differences reaching p<0.05. The distribution of explanatory variables was also similar across groups.

Table 1 Sample characteristics for 6-month Bayley outcomes and key explanatory variables, by group

| **Variable** | **Class** | **Control (N=107)** | ***F*_ENO_ (N=113)** | **Total (N=220)** | **P-value** |
| --- | --- | --- | --- | --- | --- |
| Smoker | No | 97 (91%) | 101 (89%) | 198 (90%) | 0.82 |
|  | Yes | 10 (9.3%) | 12 (11%) | 22 (10%) |  |
| SEIFA quintile | 1.0 | 10 (9.4%) | 13 (12%) | 23 (11%) | 0.28 |
|  | 2.0 | 20 (19%) | 16 (15%) | 36 (17%) |  |
|  | 3.0 | 43 (41%) | 57 (52%) | 100 (46%) |  |
|  | 4.0 | 32 (30%) | 24 (22%) | 56 (26%) |  |
|  | 5.0 | 1 (0.9%) |  | 1 (0.5%) |  |
|  | Missing | 1 | 3 | 4 |  |
| Preterm birth | No | 97 (91%) | 100 (88%) | 197 (90%) | 0.66 |
|  | Yes | 10 (9.3%) | 13 (12%) | 23 (10%) |  |
| Birthweight (g) | n | 107 | 113 | 220 |  |
|  | mean (SD) | 3397.9 (538.8) | 3311.7 (578.5) | 3353.6 (559.9) | 0.25 |
| Gestational age (w) | n | 107 | 113 | 220 |  |
|  | mean (SD) | 38.9 (1.7) | 38.8 (1.7) | 38.8 (1.7) | 0.62 |
| Infant sex | Male | 56 (52%) | 56 (50%) | 112 (51%) | 0.69 |
|  | Female | 51 (48%) | 57 (50%) | 108 (49%) |  |
| Breastfed at 6w | No | 20 (26%) | 20 (26%) | 40 (26%) | 1.00 |
|  | Yes | 58 (74%) | 56 (74%) | 114 (74%) |  |
|  | Missing | 29 | 37 | 66 |  |
| Cognitive (continuous) | n | 54 | 62 | 116 |  |
|  | mean (SD) | 10.7 (2.5) | 10.9 (2.4) | 10.8 (2.4) | 0.63 |
| Receptive language (continuous) | n | 53 | 62 | 115 |  |
|  | mean (SD) | 7.8 (1.8) | 7.9 (1.7) | 7.9 (1.7) | 0.86 |
| Expressive language (continuous) | n | 53 | 62 | 115 |  |
|  | mean (SD) | 6.8 (2.3) | 6.5 (1.7) | 6.7 (2.0) | 0.52 |
| Fine motor (continuous) | n | 52 | 61 | 113 |  |
|  | mean (SD) | 9.0 (1.5) | 8.8 (1.7) | 8.9 (1.6) | 0.40 |
| Gross motor (continuous) | n | 53 | 61 | 114 |  |
|  | mean (SD) | 9.0 (1.9) | 8.9 (2.2) | 8.9 (2.0) | 0.84 |
| Social-Emotional Composite | n | 41 | 45 | 86 |  |
|  | mean (SD) | 105.0 (12.7) | 104.8 (17.9) | 104.9 (15.6) | 0.95 |
| General Adaptive Composite | n | 40 | 37 | 77 |  |
|  | mean (SD) | 108.8 (13.7) | 113.2 (9.6) | 110.9 (12.0) | 0.11 |
| Adaptive Behaviour: Conceptual | n | 40 | 38 | 78 |  |
|  | mean (SD) | 106.3 (14.3) | 110.2 (11.9) | 108.2 (13.2) | 0.20 |
| Adaptive Behaviour: Social | n | 40 | 38 | 78 |  |
|  | mean (SD) | 111.7 (10.9) | 114.8 (8.2) | 113.2 (9.7) | 0.16 |
| Adaptive Behaviour: Practical | n | 40 | 38 | 78 |  |
|  | mean (SD) | 105.4 (12.4) | 107.3 (9.7) | 106.3 (11.1) | 0.45 |
| Cognitive (categories) | At risk | 1 (1.9%) |  | 1 (0.9%) | 0.40 |
|  | Emerging | 3 (5.6%) | 6 (9.7%) | 9 (7.8%) |  |
|  | Competent | 50 (93%) | 56 (90%) | 106 (91%) |  |
|  | Missing | 53 | 51 | 104 |  |
| Receptive language (categories) | At risk | 1 (1.9%) |  | 1 (0.9%) | 0.37 |
|  | Emerging | 10 (19%) | 16 (26%) | 26 (23%) |  |
|  | Competent | 42 (79%) | 46 (74%) | 88 (77%) |  |
|  | Missing | 54 | 51 | 105 |  |
| Expressive language (categories) | Emerging | 10 (19%) | 10 (16%) | 20 (17%) | 0.81 |
|  | Competent | 43 (81%) | 52 (84%) | 95 (83%) |  |
|  | Missing | 54 | 51 | 105 |  |
| Fine motor (categories) | At risk | 1 (1.9%) | 1 (1.6%) | 2 (1.8%) | 0.48 |
|  | Emerging | 3 (5.8%) | 8 (13%) | 11 (9.7%) |  |
|  | Competent | 48 (92%) | 52 (85%) | 100 (88%) |  |
|  | Missing | 55 | 52 | 107 |  |
| Gross motor (categories) | At risk | 1 (1.9%) | 4 (6.6%) | 5 (4.4%) | 0.50 |
|  | Emerging | 15 (28%) | 18 (30%) | 33 (29%) |  |
|  | Competent | 37 (70%) | 39 (64%) | 76 (67%) |  |
|  | Missing | 54 | 52 | 106 |  |

## 12-month developmental (Bayley) outcomes

For 12-month outcome variables (Table 2), distributions were also similar across the two groups, with no differences reaching p<0.05. The distribution of explanatory variables was also similar across groups.

Table 2 Sample characteristics for 12-month Bayley outcomes and key explanatory variables, by group

| **Variable** | **Class** | **Control (N=107)** | ***F*_ENO_ (N=113)** | **Total (N=220)** | **P-value** |
| --- | --- | --- | --- | --- | --- |
| Smoker | No | 97 (91%) | 101 (89%) | 198 (90%) | 0.82 |
|  | Yes | 10 (9.3%) | 12 (11%) | 22 (10%) |  |
| SEIFA quintile | 1.0 | 10 (9.4%) | 13 (12%) | 23 (11%) | 0.28 |
|  | 2.0 | 20 (19%) | 16 (15%) | 36 (17%) |  |
|  | 3.0 | 43 (41%) | 57 (52%) | 100 (46%) |  |
|  | 4.0 | 32 (30%) | 24 (22%) | 56 (26%) |  |
|  | 5.0 | 1 (0.9%) |  | 1 (0.5%) |  |
|  | Missing | 1 | 3 | 4 |  |
| Birthweight (g) | n | 107 | 113 | 220 |  |
|  | mean (SD) | 3397.9 (538.8) | 3311.7 (578.5) | 3353.6 (559.9) | 0.25 |
| Gestational age (w) | n | 107 | 113 | 220 |  |
|  | mean (SD) | 38.9 (1.7) | 38.8 (1.7) | 38.8 (1.7) | 0.62 |
| Infant sex | Male | 56 (52%) | 56 (50%) | 112 (51%) | 0.69 |
|  | Female | 51 (48%) | 57 (50%) | 108 (49%) |  |
| Preterm birth | No | 97 (91%) | 100 (88%) | 197 (90%) | 0.66 |
|  | Yes | 10 (9.3%) | 13 (12%) | 23 (10%) |  |
| Cognitive Composite | n | 47 | 75 | 122 |  |
|  | mean (SD) | 108.9 (11.1) | 108.5 (12.8) | 108.6 (12.1) | 0.84 |
| Language Composite | n | 47 | 74 | 121 |  |
|  | mean (SD) | 95.9 (11.7) | 95.6 (10.0) | 95.7 (10.6) | 0.90 |
| Motor Composite | n | 47 | 74 | 121 |  |
|  | mean (SD) | 97.2 (12.8) | 97.9 (12.1) | 97.6 (12.3) | 0.78 |
| Social-Emotional Composite | n | 26 | 51 | 77 |  |
|  | mean (SD) | 99.1 (19.9) | 104.7 (17.3) | 102.8 (18.3) | 0.21 |
| General Adaptive Composite | n | 21 | 41 | 62 |  |
|  | mean (SD) | 105.9 (13.9) | 104.6 (11.0) | 105.1 (12.0) | 0.70 |
| Adaptive Behaviour: Conceptual | n | 23 | 43 | 66 |  |
|  | mean (SD) | 107.1 (13.6) | 105.7 (11.8) | 106.2 (12.3) | 0.65 |
| Adaptive Behaviour: Social | n | 23 | 47 | 70 |  |
|  | mean (SD) | 110.4 (15.7) | 111.8 (10.0) | 111.3 (12.1) | 0.66 |
| Adaptive Behaviour: Practical | n | 23 | 42 | 65 |  |
|  | mean (SD) | 99.0 (12.7) | 96.5 (10.9) | 97.4 (11.5) | 0.39 |

# Intervention effect estimates for 12-month developmental outcomes.

## Primary developmental (Bayley) outcomes at 12 months

Table 3 Effect estimates for Bayley 12-month primary outcomes (sensitivity analysis).

| **Outcome** | **Control mean (SD)** | ***F*_ENO_ mean (SD)** | **Coefficient (95% CI) *F*_ENO_ - Control** | **P Value** |
| --- | --- | --- | --- | --- |
| Cognitive Composite | 108.9 (11.1) | 108.5 (12.8) | 0.2 (-5.1, 5.6) | 0.93 |
| Language Composite | 95.9 (11.7) | 95.6 (10.0) | -0.5 (-6.0, 5.1) | 0.87 |
| Motor Composite | 97.2 (12.8) | 97.9 (12.1) | 3.5 (-2.4, 9.4) | 0.25 |

## Secondary developmental (Bayley) outcomes at 12 months

Table 4 Effect estimates for Bayley 12-month secondary outcomes.

| **Outcome** | **Control mean (SD)** | ***F*_ENO_ mean (SD)** | **Coefficient (95% CI) *F*_ENO_ - Control** | **P Value** |
| --- | --- | --- | --- | --- |
| Social-Emotional Composite | 99.1 (19.9) | 104.7 (17.3) | 8.6 (-2.4, 19.6) | 0.13 |
| General Adaptive Composite (GAC) | 105.9 (13.9) | 104.6 (11.0) | 1.5 (-5.8, 8.7) | 0.70 |
| Adaptive Behaviour: Conceptual | 107.1 (13.6) | 105.7 (11.8) | -1.0 (-8.3, 6.3) | 0.79 |
| Adaptive Behaviour: Social | 110.4 (15.7) | 111.8 (10.0) | 6.0 (-1.3, 13.4) | 0.11 |
| Adaptive Behaviour: Practical | 99.0 (12.7) | 96.5 (10.9) | 1.6 (-5.6, 8.8) | 0.67 |

# Intervention effect estimates for 6-month developmental outcomes

## Continuous developmental (Bayley) outcomes at 6 months

Most continuous outcomes showed negligible differences between groups with point estimates close to 0, confidence intervals spanning 0 and p-values being non-significant (Table 7). The difference between infants of FENO and Control mothers reached p=0.05 for the outcome of General Adaptive Composite (GAC), with a higher mean GAC score in infants of mothers randomised to FENO (113.2 vs 108.8). Results were similar in sensitivity analyses adjusting for additional potential confounding variables (Table 8).

Table 5 Effect estimates for Bayley 6-month outcomes (continuous outcomes)

| **Outcome** | **Control mean (SD)** | ***F*_ENO_ mean (SD)** | **Coefficient (95% CI) *F*_ENO_ - Control** | **P Value** |
| --- | --- | --- | --- | --- |
| Cognitive | 10.7 (2.5) | 10.9 (2.4) | 0.5 (-0.4, 1.5) | 0.29 |
| Receptive language | 7.8 (1.8) | 7.9 (1.7) | 0.3 (-0.4, 1.0) | 0.33 |
| Expressive language | 6.8 (2.3) | 6.5 (1.7) | 0.1 (-0.8, 1.0) | 0.82 |
| Fine motor | 9.0 (1.5) | 8.8 (1.7) | -0.2 (-0.8, 0.4) | 0.48 |
| Gross motor | 9.0 (1.9) | 8.9 (2.2) | 0.2 (-0.6, 1.0) | 0.64 |
| Social-Emotional Composite | 105.0 (12.7) | 104.8 (17.9) | 0.9 (-7.4, 9.2) | 0.83 |
| General Adaptive Composite (GAC) | 108.8 (13.7) | 113.2 (9.6) | 2.7 (-3.2, 8.6) | 0.37 |
| Adaptive Behaviour: Conceptual | 106.3 (14.3) | 110.2 (11.9) | 0.4 (-5.9, 6.7) | 0.90 |
| Adaptive Behaviour: Social | 111.7 (10.9) | 114.8 (8.2) | 3.2 (-1.6, 8.0) | 0.20 |
| Adaptive Behaviour: Practical | 105.4 (12.4) | 107.3 (9.7) | -0.2 (-6.1, 5.7) | 0.95 |

Bayley-III categorical outcomes at 6-months were modelled using logistic regression. The modelled outcome was binary: “Competent”, which was compared to “At risk/emerging”. Table 6 displays the frequency of infants in the “Competent” category, and odds ratios represent the multiplicative increase in the odds of being “Competent” for infants of *F*_ENO_ mothers vs infants of Control mothers.

The frequency of “Competent” was high for all outcomes, and no group differences were apparent, with confidence intervals for all odds ratios spanning 1, and p-values>0.05. All Bayes Factors were between 3 and 20, providing “positive” evidence for the null hypothesis of no intervention effect for all outcomes. The posterior probability of H_0_ was above 0.84 for all outcomes, providing further support for all null hypotheses. Results were similar for the model adjusted only for smoking (Table 6) and the model adjusted for additional potential confounding variables (Table 6 – Supplementary data).

Table 6. Effect estimates for Bayley at 6- months (categorical)

| **Outcome** | **Control Frequency/**  **Total (%)** | ***F*_ENO_ Frequency/**  **Total (%)** | **Odds Ratio (95% CI) FENO vs Control** | **P Value** | **Bayes Factor** | **Posterior probability of H_0_** |
| --- | --- | --- | --- | --- | --- | --- |
| Cognitive | 50/54 (92.6) | 56/62 (90.3) | 0.75 (0.20, 2.81) | 0.67 | 9.80 | 0.91 |
| Receptive language | 42/53 (79.2) | 46/62 (74.2) | 0.75 (0.31, 1.79) | 0.51 | 8.64 | 0.90 |
| Expressive language | 43/53 (81.1) | 52/62 (83.9) | 1.21 (0.46, 3.17) | 0.70 | 9.97 | 0.91 |
| Fine motor | 48/52 (92.3) | 52/61 (85.2) | 0.48 (0.14, 1.67) | 0.25 | 5.26 | 0.84 |
| Gross motor | 37/53 (69.8) | 39/61 (63.9) | 0.78 (0.36, 1.72) | 0.54 | 8.87 | 0.90 |

## Binary developmental (Bayley) outcomes at 6 months

**Table 7 Effect estimates for Bayley 6-month outcomes (categorical outcomes)**

| **Outcome** | **Control Frequency/**  **Total (%)** | ***F*_ENO_ Frequency/**  **Total (%)** | **Odds Ratio (95% CI) *F*_ENO_ vs Control** | **P Value** |
| --- | --- | --- | --- | --- |
| Cognitive | 50/54 (92.6) | 56/62 (90.3) | 1.01 (0.12, 8.73) | 0.99 |
| Receptive language | 42/53 (79.2) | 46/62 (74.2) | 1.78 (0.47, 6.76) | 0.40 |
| Expressive language | 43/53 (81.1) | 52/62 (83.9) | 2.82 (0.64, 12.3) | 0.17 |
| Fine motor | 48/52 (92.3) | 52/61 (85.2) | 0.49 (0.08, 3.06) | 0.44 |
| Gross motor | 37/53 (69.8) | 39/61 (63.9) | 0.98 (0.35, 2.76) | 0.97 |

# Intervention effect estimates for temperament outcomes

Table 8 Effect estimates for temperament outcomes (continuous outcomes)

| **Outcome** | **Control mean (SD)** | ***F*_ENO_ mean (SD)** | **Coefficient (95% CI) *F*_ENO_ - Control** | **P Value** | **Bayes Factor** | **Posterior probability of H_0_** |
| --- | --- | --- | --- | --- | --- | --- |
| 6 weeks |  |  |  |  |  |  |
| Activity | 3.7 (0.6) | 3.6 (0.7) | -0.1 (-0.3, 0.1) | 0.30 | 6.77 | 0.87 |
| Rhythmicity | 3.4 (0.8) | 3.3 (0.7) | -0.0 (-0.3, 0.2) | 0.71 | 11.23 | 0.92 |
| Approach | 2.5 (0.8) | 2.5 (0.6) | -0.0 (-0.2, 0.2) | 0.91 | 11.97 | 0.92 |
| Adaptability | 2.3 (0.6) | 2.3 (0.7) | -0.0 (-0.3, 0.2) | 0.76 | 10.93 | 0.92 |
| Intensity | 3.9 (0.9) | 3.7 (0.9) | -0.2 (-0.5, 0.1) | 0.12 | 3.69 | 0.79 |
| Mood | 2.8 (0.7) | 2.9 (0.8) | 0.1 (-0.2, 0.3) | 0.60 | 10.40 | 0.91 |
| Persistence | 2.7 (0.9) | 2.7 (0.9) | 0.0 (-0.3, 0.3) | 0.95 | 11.81 | 0.92 |
| Distractibility | 2.3 (0.7) | 2.3 (0.8) | 0.0 (-0.2, 0.3) | 0.82 | 11.52 | 0.92 |
| Threshold | 4.3 (0.7) | 4.3 (0.6) | 0.0 (-0.2, 0.2) | 0.99 | 11.96 | 0.92 |
| 6 months |  |  |  |  |  |  |
| Activity | 4.2 (0.5) | 4.3 (0.5) | 0.0 (-0.2, 0.2) | 0.80 | 8.98 | 0.90 |
| Rhythmicity | 2.8 (0.8) | 2.8 (0.8) | -0.0 (-0.4, 0.3) | 0.79 | 8.91 | 0.90 |
| Approach | 2.4 (0.7) | 2.4 (0.6) | -0.1 (-0.4, 0.2) | 0.51 | 7.37 | 0.88 |
| Adaptability | 2.1 (0.6) | 2.2 (0.5) | 0.1 (-0.2, 0.3) | 0.61 | 8.08 | 0.89 |
| Intensity | 3.6 (0.6) | 3.6 (0.6) | 0.1 (-0.2, 0.3) | 0.56 | 7.84 | 0.89 |
| Mood | 2.6 (0.6) | 2.8 (0.7) | 0.1 (-0.2, 0.4) | 0.42 | 6.75 | 0.87 |
| Persistence | 3.1 (0.9) | 3.1 (0.7) | -0.0 (-0.4, 0.3) | 0.80 | 9.03 | 0.90 |
| Distractibility | 2.2 (0.6) | 2.2 (0.7) | -0.1 (-0.3, 0.2) | 0.61 | 8.19 | 0.89 |
| Threshold | 3.9 (0.6) | 3.8 (0.5) | -0.0 (-0.3, 0.2) | 0.85 | 9.01 | 0.90 |
| 12 months |  |  |  |  |  |  |
| Activity | 3.9 (0.7) | 3.9 (0.6) | -0.0 (-0.3, 0.3) | 0.97 | 8.77 | 0.90 |
| Rhythmicity | 2.7 (0.9) | 2.6 (0.8) | -0.1 (-0.4, 0.3) | 0.75 | 8.59 | 0.90 |
| Approach | 3.0 (0.9) | 3.0 (0.8) | -0.0 (-0.4, 0.4) | 0.98 | 8.94 | 0.90 |
| Adaptability | 3.6 (0.8) | 3.5 (0.7) | -0.1 (-0.5, 0.2) | 0.39 | 6.05 | 0.86 |
| Intensity | 3.8 (0.6) | 3.8 (0.5) | -0.0 (-0.3, 0.2) | 0.92 | 8.89 | 0.90 |
| Mood | 3.1 (0.7) | 3.2 (0.6) | 0.1 (-0.2, 0.4) | 0.59 | 7.73 | 0.89 |
| Persistence | 3.8 (0.7) | 3.8 (0.7) | -0.0 (-0.3, 0.3) | 0.85 | 8.78 | 0.90 |
| Distractibility | 4.4 (0.6) | 4.4 (0.5) | 0.0 (-0.2, 0.3) | 0.70 | 8.37 | 0.89 |
| Threshold | 3.6 (0.6) | 3.5 (0.7) | -0.1 (-0.4, 0.2) | 0.44 | 6.62 | 0.87 |

Table 9 Effect estimates for temperament outcomes (continuous outcomes) – sensitivity analysis

| **Outcome** | **Control mean (SD)** | ***F*_ENO_ mean (SD)** | **Coefficient (95% CI) *F*_ENO_ - Control** | **P Value** |
| --- | --- | --- | --- | --- |
| 6 weeks |  |  |  |  |
| Activity | 3.7 (0.6) | 3.6 (0.7) | -0.1 (-0.3, 0.1) | 0.36 |
| Rhythmicity | 3.4 (0.8) | 3.3 (0.7) | -0.1 (-0.3, 0.2) | 0.55 |
| Approach | 2.5 (0.8) | 2.5 (0.6) | 0.0 (-0.2, 0.2) | 0.95 |
| Adaptability | 2.3 (0.6) | 2.3 (0.7) | -0.0 (-0.2, 0.2) | 0.85 |
| Intensity | 3.9 (0.9) | 3.7 (0.9) | -0.2 (-0.5, 0.1) | 0.13 |
| Mood | 2.8 (0.7) | 2.9 (0.8) | 0.0 (-0.2, 0.3) | 0.80 |
| Persistence | 2.7 (0.9) | 2.7 (0.9) | -0.0 (-0.3, 0.3) | 0.89 |
| Distractibility | 2.3 (0.7) | 2.3 (0.8) | 0.0 (-0.2, 0.3) | 0.77 |
| Threshold | 4.3 (0.7) | 4.3 (0.6) | 0.0 (-0.2, 0.2) | 0.98 |
| 6 months |  |  |  |  |
| Activity | 4.2 (0.5) | 4.3 (0.5) | 0.1 (-0.1, 0.4) | 0.24 |
| Rhythmicity | 2.8 (0.8) | 2.8 (0.8) | -0.1 (-0.4, 0.3) | 0.73 |
| Approach | 2.4 (0.7) | 2.4 (0.6) | 0.1 (-0.2, 0.4) | 0.54 |
| Adaptability | 2.1 (0.6) | 2.2 (0.5) | 0.1 (-0.2, 0.4) | 0.52 |
| Intensity | 3.6 (0.6) | 3.6 (0.6) | 0.1 (-0.2, 0.4) | 0.39 |
| Mood | 2.6 (0.6) | 2.8 (0.7) | 0.4 (0.0, 0.7) | 0.03 |
| Persistence | 3.1 (0.9) | 3.1 (0.7) | -0.1 (-0.5, 0.3) | 0.60 |
| Distractibility | 2.2 (0.6) | 2.2 (0.7) | -0.1 (-0.4, 0.3) | 0.72 |
| Threshold | 3.9 (0.6) | 3.8 (0.5) | -0.1 (-0.3, 0.2) | 0.70 |
| 12 months |  |  |  |  |
| Activity | 3.9 (0.7) | 3.9 (0.6) | 0.0 (-0.3, 0.4) | 0.82 |
| Rhythmicity | 2.7 (0.9) | 2.6 (0.8) | -0.3 (-0.7, 0.2) | 0.19 |
| Approach | 3.0 (0.9) | 3.0 (0.8) | 0.0 (-0.5, 0.6) | 0.93 |
| Adaptability | 3.6 (0.8) | 3.5 (0.7) | -0.3 (-0.8, 0.1) | 0.13 |
| Intensity | 3.8 (0.6) | 3.8 (0.5) | -0.0 (-0.4, 0.3) | 0.84 |
| Mood | 3.1 (0.7) | 3.2 (0.6) | 0.2 (-0.2, 0.5) | 0.39 |
| Persistence | 3.8 (0.7) | 3.8 (0.7) | 0.0 (-0.3, 0.4) | 0.99 |
| Distractibility | 4.4 (0.6) | 4.4 (0.5) | 0.2 (-0.1, 0.5) | 0.16 |
| Threshold | 3.6 (0.6) | 3.5 (0.7) | -0.0 (-0.5, 0.4) | 0.81 |

# Intervention effect estimates for sensory processing outcomes

Table 10 Effect estimates for sensory processing outcomes (continuous outcomes)

| **Outcome** | **Control mean (SD)** | ***F*_ENO_ mean (SD)** | **Coefficient (95% CI) *F*_ENO_ - Control** | **P Value** | **Bayes Factor** | **Posterior probability of H_0_** |
| --- | --- | --- | --- | --- | --- | --- |
| 6 weeks |  |  |  |  |  |  |
| General Processing | 17.6 (3.5) | 17.4 (3.3) | -0.2 (-1.4, 0.9) | 0.68 | 10.69 | 0.91 |
| Auditory Processing | 10.2 (2.6) | 10.5 (2.2) | 0.3 (-0.5, 1.1) | 0.48 | 9.21 | 0.90 |
| Visual Processing | 6.8 (2.2) | 6.8 (2.3) | 0.0 (-0.7, 0.8) | 0.97 | 11.87 | 0.92 |
| Touch Processing | 5.3 (2.5) | 5.6 (2.1) | 0.4 (-0.4, 1.1) | 0.35 | 7.67 | 0.88 |
| Movement Processing | 8.9 (2.0) | 8.6 (1.7) | -0.2 (-0.8, 0.4) | 0.44 | 8.84 | 0.90 |
| Oral Sensory | 5.3 (1.4) | 5.3 (1.4) | 0.0 (-0.4, 0.5) | 0.97 | 11.91 | 0.92 |
| Total Processing | 53.9 (6.9) | 54.2 (6.0) | 0.3 (-1.9, 2.5) | 0.79 | 11.14 | 0.92 |
| 6 months |  |  |  |  |  |  |
| General Processing | 16.1 (3.6) | 15.8 (3.7) | -0.3 (-1.8, 1.2) | 0.71 | 8.74 | 0.90 |
| Auditory Processing | 12.0 (1.7) | 11.7 (1.7) | -0.3 (-1.0, 0.4) | 0.44 | 7.05 | 0.88 |
| Visual Processing | 6.4 (2.5) | 6.0 (2.3) | -0.4 (-1.4, 0.7) | 0.49 | 7.35 | 0.88 |
| Touch Processing | 5.4 (2.3) | 5.2 (2.2) | -0.1 (-1.1, 0.8) | 0.75 | 9.08 | 0.90 |
| Movement Processing | 8.5 (2.2) | 8.2 (2.1) | -0.3 (-1.2, 0.6) | 0.50 | 7.59 | 0.88 |
| Oral Sensory | 6.0 (0.5) | 5.6 (1.1) | -0.4 (-0.7, -0.0) | 0.04 | 1.18 | 0.54 |
| Total Processing | 54.1 (7.3) | 53.0 (7.9) | -1.0 (-4.3, 2.2) | 0.54 | 7.62 | 0.88 |
| 12 months |  |  |  |  |  |  |
| General Processing | 16.5 (6.1) | 16.8 (5.0) | 0.3 (-2.2, 2.7) | 0.84 | 8.81 | 0.90 |
| Auditory Processing | 10.5 (4.0) | 10.3 (3.0) | -0.2 (-1.8, 1.3) | 0.79 | 8.65 | 0.90 |
| Visual Processing | 16.8 (3.6) | 16.8 (3.7) | -0.0 (-1.7, 1.6) | 0.97 | 8.88 | 0.90 |
| Touch Processing | 9.9 (2.8) | 10.7 (2.9) | 0.8 (-0.5, 2.2) | 0.20 | 4.05 | 0.80 |
| Movement Processing | 19.0 (2.3) | 18.3 (3.0) | -0.8 (-2.0, 0.5) | 0.21 | 4.14 | 0.81 |
| Oral Sensory Processing | 12.8 (3.7) | 11.7 (3.9) | -1.1 (-2.9, 0.6) | 0.21 | 4.04 | 0.80 |
| Behavioural Processing | 11.2 (4.0) | 11.1 (3.2) | -0.1 (-1.7, 1.6) | 0.94 | 8.87 | 0.90 |
| Sensation Seeking Quadrant | 31.0 (3.4) | 30.0 (4.0) | -1.0 (-2.7, 0.8) | 0.28 | 5.00 | 0.83 |
| Sensation Avoidance Quadrant | 15.9 (4.4) | 15.3 (3.8) | -0.6 (-2.4, 1.3) | 0.54 | 7.39 | 0.88 |
| Sensation Sensitivity Quadrant | 24.0 (6.9) | 24.4 (5.7) | 0.5 (-2.4, 3.4) | 0.74 | 8.26 | 0.89 |
| Low Registration Quadrant | 16.3 (4.5) | 15.7 (4.0) | -0.6 (-2.6, 1.3) | 0.52 | 7.22 | 0.88 |

Table 11 Effect estimates for sensory processing outcomes (continuous outcomes) – sensitivity analysis

| **Outcome** | **Control mean (SD)** | ***F*_ENO_ mean (SD)** | **Coefficient (95% CI) *F*_ENO_ - Control** | **P Value** |
| --- | --- | --- | --- | --- |
| 6 weeks |  |  |  |  |
| General Processing | 17.6 (3.5) | 17.4 (3.3) | -0.3 (-1.5, 0.9) | 0.61 |
| Auditory Processing | 10.2 (2.6) | 10.5 (2.2) | 0.3 (-0.5, 1.1) | 0.45 |
| Visual Processing | 6.8 (2.2) | 6.8 (2.3) | 0.1 (-0.6, 0.9) | 0.78 |
| Touch Processing | 5.3 (2.5) | 5.6 (2.1) | 0.3 (-0.4, 1.1) | 0.41 |
| Movement Processing | 8.9 (2.0) | 8.6 (1.7) | -0.2 (-0.9, 0.4) | 0.43 |
| Oral Sensory | 5.3 (1.4) | 5.3 (1.4) | 0.0 (-0.5, 0.5) | 0.98 |
| Total Processing | 53.9 (6.9) | 54.2 (6.0) | 0.3 (-2.0, 2.5) | 0.82 |
| 6 months |  |  |  |  |
| General Processing | 16.1 (3.6) | 15.8 (3.7) | -0.2 (-2.1, 1.6) | 0.82 |
| Auditory Processing | 12.0 (1.7) | 11.7 (1.7) | -0.6 (-1.5, 0.4) | 0.24 |
| Visual Processing | 6.4 (2.5) | 6.0 (2.3) | -0.3 (-1.6, 1.1) | 0.69 |
| Touch Processing | 5.4 (2.3) | 5.2 (2.2) | 0.3 (-0.8, 1.4) | 0.58 |
| Movement Processing | 8.5 (2.2) | 8.2 (2.1) | -0.1 (-1.0, 0.8) | 0.83 |
| Oral Sensory | 6.0 (0.5) | 5.6 (1.1) | -0.2 (-0.7, 0.3) | 0.40 |
| Total Processing | 54.1 (7.3) | 53.0 (7.9) | -1.1 (-5.0, 2.7) | 0.57 |
| 12 months |  |  |  |  |
| General Processing | 16.5 (6.1) | 16.8 (5.0) | -0.1 (-3.5, 3.3) | 0.97 |
| Auditory Processing | 10.5 (4.0) | 10.3 (3.0) | -0.3 (-2.6, 2.0) | 0.79 |
| Visual Processing | 16.8 (3.6) | 16.8 (3.7) | 0.4 (-1.8, 2.6) | 0.75 |
| Touch Processing | 9.9 (2.8) | 10.7 (2.9) | 1.7 (0.1, 3.4) | 0.04 |
| Movement Processing | 19.0 (2.3) | 18.3 (3.0) | 0.3 (-1.2, 1.7) | 0.72 |
| Oral Sensory Processing | 12.8 (3.7) | 11.7 (3.9) | -0.0 (-2.3, 2.2) | 0.98 |
| Behavioural Processing | 11.2 (4.0) | 11.1 (3.2) | 0.5 (-1.6, 2.6) | 0.66 |
| Sensation Seeking Quadrant | 31.0 (3.4) | 30.0 (4.0) | -0.2 (-2.2, 1.9) | 0.87 |
| Sensation Avoidance Quadrant | 15.9 (4.4) | 15.3 (3.8) | 0.7 (-1.7, 3.1) | 0.55 |
| Sensation Sensitivity Quadrant | 24.0 (6.9) | 24.4 (5.7) | 0.9 (-3.0, 4.9) | 0.64 |
| Low Registration Quadrant | 16.3 (4.5) | 15.7 (4.0) | 0.4 (-2.4, 3.3) | 0.77 |

Table 12 Effect estimates for autism (FYI) outcomes – continuous outcomes

| **Outcome** | **Control mean (SD)** | ***F*_ENO_ mean (SD)** | **Coefficient (95% CI) *F*_ENO_ - Control** | **P Value** | **Bayes Factor** | **Posterior probability of H_0_** |
| --- | --- | --- | --- | --- | --- | --- |
| Social communication domain score | 9.21 (11.3) | 8.21 (8.3) | -1.04 (-5.16, 3.07) | 0.62 | 7.12 | 0.88 |
| Sensory regulatory domain score | 9.77 (10.3) | 9.13 (8.0) | -0.64 (-4.51, 3.24) | 0.75 | 8.18 | 0.89 |
| Total score | 9.49 (9.6) | 8.67 (6.4) | -0.84 (-4.20, 2.52) | 0.62 | 8.5 | 0.89 |

Table 13 Effect estimates for autism (FYI) outcomes (binary outcomes)

| **Outcome** | **Control Frequency/**  **Total (%)** | ***F*_ENO_ Frequency/**  **Total (%)** | **Odds Ratio (95% CI) *F*_ENO_ vs Control** | **P Value** | **Bayes Factor** | **Posterior probability of H_0_** |
| --- | --- | --- | --- | --- | --- | --- |
| Autism likelihood high  (social communication score ≥22.5) | 4/31 (12.9) | 5/58 (8.6) | 0.63 (0.15, 2.54) | 0.51 | 7.76 | 0.89 |
| Autism likelihood high  (sensory regulation score ≥14.75) | 7/31 (22.6) | 13/58 (22.4) | 1.00 (0.35, 2.86) | 1.0 | 9.43 | 0.90 |
| Autism likelihood high  (FYI total score ≥19.2) | 5/31 (16.1) | 5/58 (8.6) | 0.48 (0.13, 1.82) | 0.28 | 5.48 | 0.85 |


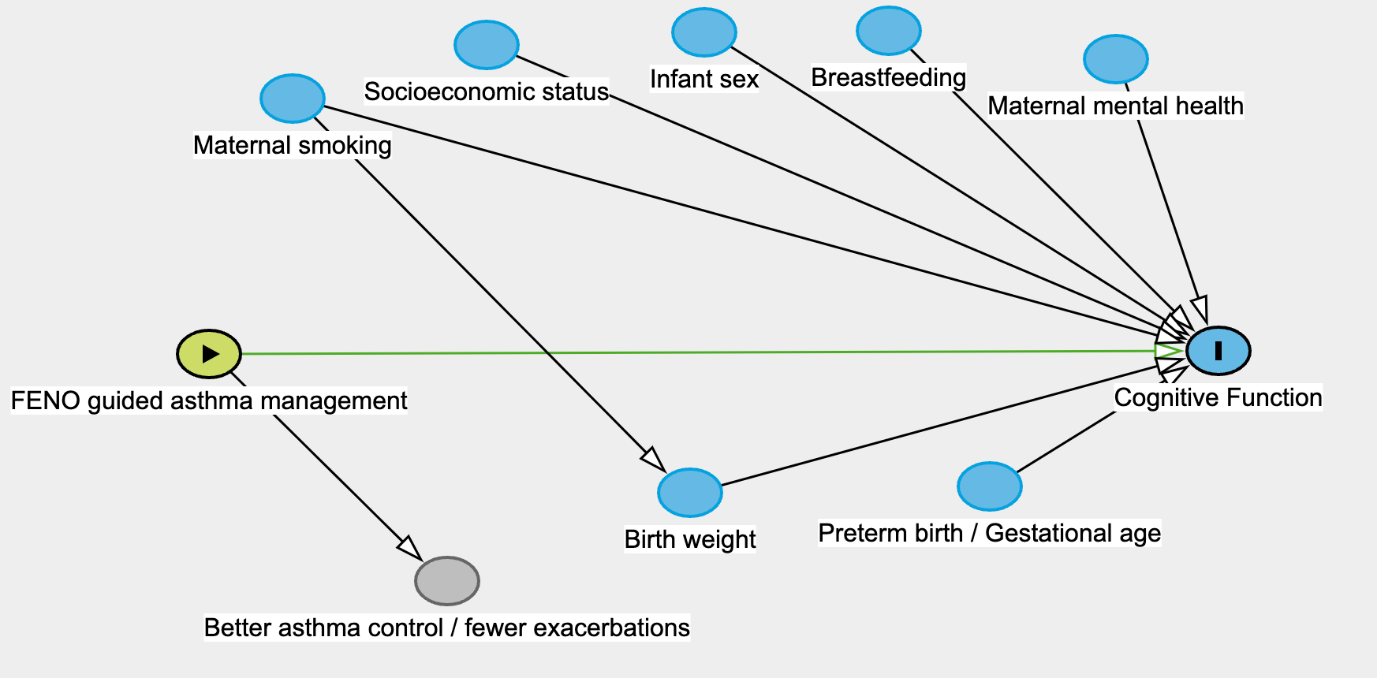


Figure S2. Directed Acyclic Graph representing the assumed causal relationships between the exposure, covariates, and the outcome(s). Developed using DAGitty(3).

1. Turner-Brown LM, Baranek GT, Reznick JS, Watson LR, Crais ER. The First Year Inventory: A longitudinal follow-up of 12-month-old to 3-year-old children. Autism. 2013;17(5):527-40.

2. Bayley N. Bayley scales of infant and toddler development. 2006.

3. Textor J, Hardt J, Knüppel S. DAGitty: a graphical tool for analyzing causal diagrams. Epidemiology. 2011;22(5):745.
